# Deep Active Learning for Lung Disease Severity Classification from Chest X-rays: Learning with Less Data in the Presence of Class Imbalance

**DOI:** 10.1007/s10278-025-01752-8

**Published:** 2025-12-10

**Authors:** Roy M. Gabriel, Mohammadreza Zandehshahvar, Marly van Assen, Nattakorn Kittisut, Kyle Peters, Carlo N. De Cecco, Ali Adibi

**Affiliations:** 1 School of Electrical and Computer Engineering, Georgia Institute of Technology, 791 Atlantic Dr NW, Atlanta, GA 30332 USA; 2 Department of Radiology and Imaging Sciences, Emory University, Atlanta, GA USA; 3 College of Computing, Georgia Institute of Technology, Atlanta, GA USA

**Keywords:** Deep active learning, Chest X-ray classification, Lung disease severity, Bayesian Neural Networks, Class imbalance in medical imaging

## Abstract

To reduce the amount of required labeled data for lung disease severity classification from chest X-rays (CXRs) under class imbalance, this study applied deep active learning with a Bayesian Neural Network (BNN) approximation and weighted loss function. This retrospective study collected 2319 CXRs from 963 patients (mean age, 59.2 ± 16.6 years; 481 females) at Emory Healthcare affiliated hospitals between January and November 2020. All patients had clinically confirmed COVID-19. Each CXR was independently labeled by 3 to 6 board-certified radiologists as normal, moderate, or severe. A deep neural network with Monte Carlo Dropout was trained using active learning to classify disease severity. Various acquisition functions were used to iteratively select the most informative samples from an unlabeled pool. Performance was evaluated using accuracy, area under the receiver operating characteristic curve (AU-ROC), and area under the precision-recall curve (AU-PRC). Training time and acquisition time were recorded. Statistical analysis included descriptive metrics and performance comparisons across acquisition strategies. Least Confidence achieved 92.8% accuracy (AU-ROC, 0.95) in binary classification (normal vs. diseased) using 9.24% of the training data. In the multi-class setting, Mean STD achieved 70.5% accuracy (AU-ROC, 0.85) using 21.87% of the labeled data. These methods outperformed more complex and computationally expensive acquisition functions and significantly reduced labeling needs. Deep active learning with BNN approximation and weighted loss effectively reduces labeled data requirements while addressing class imbalance, maintaining or exceeding diagnostic performance.

**Supplementary Information:**

The online version contains supplementary material available at https://doi.org/10.1007/s10278-025-01752-8.

## Introduction

The increasing prevalence and detection of pulmonary diseases have highlighted the urgent need for reliable and time efficient approaches for accurate diagnosis. Among these diagnostic solutions, chest X-rays (CXRs) remain a widely used imaging modality due to their availability, low cost, and diagnostic utility. However, the surge in imaging volume, in combination with a global shortage of radiologists, has led to delays in diagnosis and treatment [1].

The development of artificial intelligence (AI)-based tools has emerged as a critical solution, particularly in the medical field [2, 3]. However, AI models are highly dependent on the availability and quality of labeled training data, which is often limited by the significant expense and expertise required for labeling medical images [4]. Given these constraints, approaches like active learning are needed to reduce the number of required labels and to focus on gathering labels for the most informative images.

Concurrently, fully supervised AI pipelines have reported strong CXR performance for lung-disease classification [5–7], and similar accuracy gains are seen in other imaging domains (e.g., otoscopy) [8]. While these models demonstrate remarkable performance, their dependence on large, expertly labeled datasets remains a major bottleneck for clinical scalability, highlighting the need for more label-efficient and adaptive AI frameworks.

To address this challenge, active learning is a machine learning technique that selectively chooses the most informative data points to train on. The key to effective active learning lies in the selection of samples, which is driven by an acquisition function. An acquisition function evaluates the informativeness of each unlabeled sample, guiding the selection process by identifying which samples, if labeled, would most improve the model. By doing this, active learning reduces the need for extensive annotated datasets while still achieving high performance [2–4, 9–11].

However, applying active learning in medical imaging presents its own set of challenges. Medical datasets are often imbalanced, where certain conditions, outcomes, or severity levels are underrepresented due to the complexity of medical images and the scarcity of data [12, 13]. This imbalance can complicate the learning process and lead to biased models that may not generalize well [14].

This study investigates the integration of deep active learning techniques with a weighted loss strategy to handle class imbalance effectively. Using CXRs for the diagnosis of COVID-19 as a case study, we evaluate various acquisition functions to identify those most effective at achieving baseline diagnostic performance with minimal labeled data. We hypothesize that combining active learning with weighted loss training, which penalizes the loss function based on inverse class label proportions, will allow us to achieve competitive diagnostic performance with significantly less labeled training data.

Unlike prior works that assume subsets of these ideas, such as balanced datasets, Bayesian uncertainty modeling, overlooking imbalance, or proposing new acquisition strategies to mitigate the imbalance [15–18], our contribution lies in unifying these elements within a single, controlled, and reproducible framework. Specifically, we integrate severe class imbalance, patient-level stratification to prevent data leakage, Bayesian Monte Carlo (MC) dropout for uncertainty estimation, and comprehensive, clinically meaningful evaluation metrics. This holistic design reflects realistic medical-imaging constraints often overlooked in previous works. The resulting pipeline provides a benchmark and practical recipe for deploying active learning in clinical AI settings where labeling cost, imbalance, and reproducibility are major bottlenecks.

## Materials and Methods

### Dataset

This retrospective study included 963 consecutive adult patients (≥ 18 years old) diagnosed with COVID-19 using real-time reverse transcriptase-polymerase chain reaction who underwent diagnostic CXRs at Emory Healthcare affiliated hospitals (Alanta, GA, USA) [19–22]. The data included patients seen between 01/30/2020 and 11/05/2020. Of the 963 patients in this study, between 396 and 658 patients were included in prior publications that focused on different objectives, such as severity scoring or radiologist-AI variability assessment [19–22]. This study expands upon that work by using a larger patient number and introducing an active learning framework to evaluate model performance across varying levels of class imbalance. The need for informed consent was waived by Emory University institutional review board. Both in-patients and out-patients were included. All CXRs acquired during the entire hospital admission of the patient were collected, ranging from 1 to 30 images per patient. Posteroanterior and anteroposterior CXR projections were obtained using standard clinical protocols. Lateral views were excluded due to limited standardization across patients. Depending on patient condition, CXRs were taken with a portable X-ray unit and in a supine, erect, or semi-erect position.

Basic demographic information, including age at the time of scan, gender, and body mass index (BMI), was collected. All patient data were de-identified according to Health Insurance Portability and Accountability Act and hospital-specific guidelines.

**Fig. 1:**
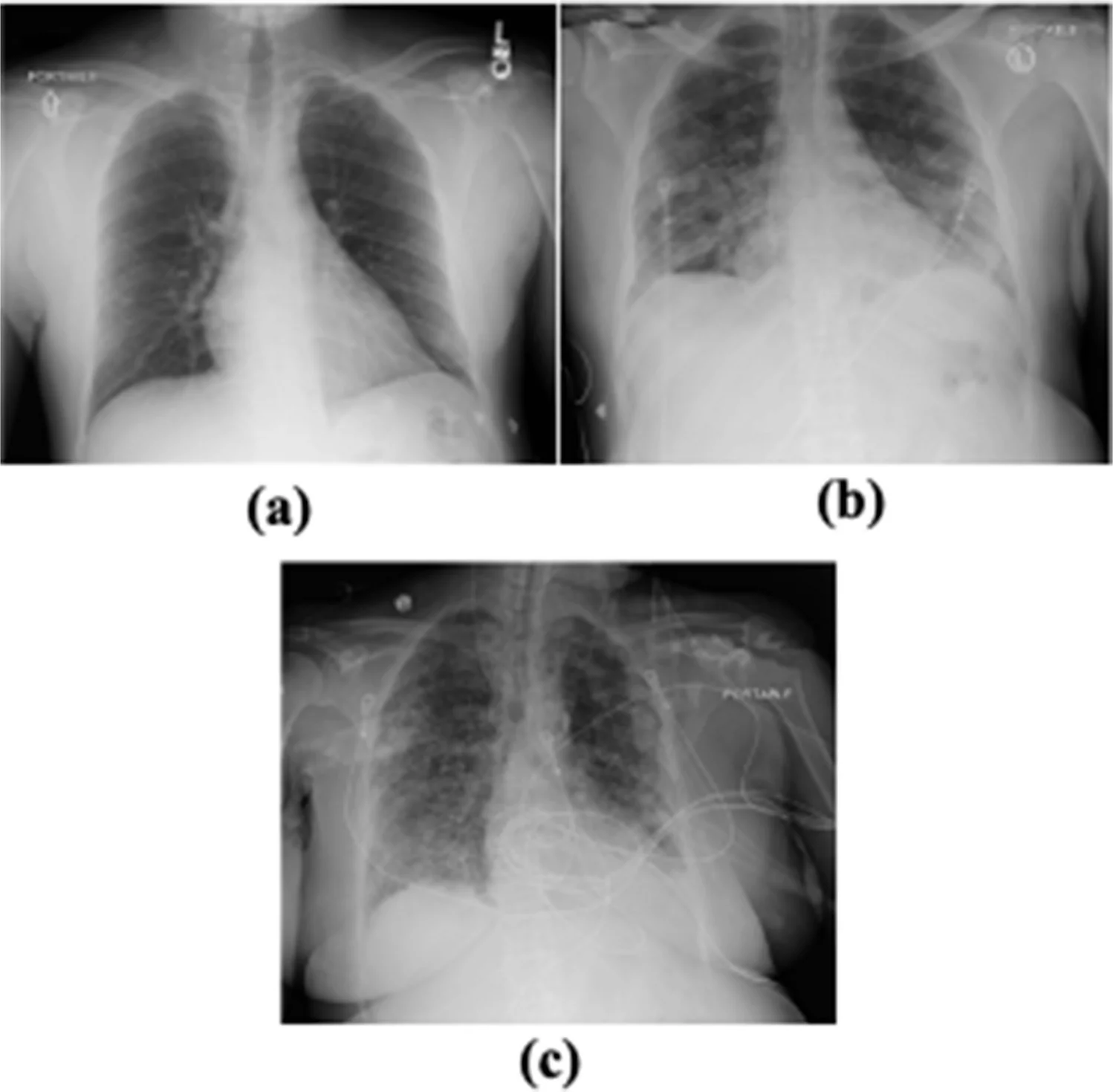
Overview of examples of different severity grades based on radiographic findings in chest X-rays: Normal (**a**): No clear sign of disease exists in the lung regions. Moderate (**b**): Bilateral opacification involving the peripheral mid and lower lung zones with approximately ≥ 25% and ≤ 50% involvement of the most severely affected lung. Severe (**c**): Patchy bilateral opacification with more than 50% involvement of the most severely affected lung

Data were labeled by three to six cardiothoracic radiologists, with experience reading CXRs ranging from 2 to 15 years. An initial batch of cases was labeled by all six experts to assess inter-reader variability, which demonstrated good agreement (Kendall’s *τ* = 0.67–0.78) [20]. Based on this consistency, subsequent batches were labeled by at least three radiologists without loss of reliability. Labels were divided into training, validation, and testing sets with a 70/10/20 split. None of the experts had insight into clinical data and/or outcomes, and the data were presented in a randomized order. Disease severity was categorized as normal, moderate, or severe according to the guidelines outlined in Fig. 1a–c. All readers used the same guidelines and were provided with examples for each class before labeling the dataset. The labeling was performed independently by the readers using an in-house designed labeling tool, which randomized the CXRs and allowed for image size adjustment for better visualization. No indeterminate results occurred. The reference standard was determined by taking the median label from the radiologists for each CXR.

To quantify label consistency, we reference the comprehensive reader-variability study by [20], which used the same multireader dataset. That work reported good inter-observer agreement among experienced radiologists (Kendall’s *τ* = 0.67–0.78) and high intra-observer consistency (*τ* = 0.77). These findings demonstrate that our median-aggregation approach provides a robust and reproducible ground truth for training and evaluation.

## Data Preprocessing and Labeling

Data preprocessing involved resizing, normalization, and augmentation of CXRs to prepare for effective model training. Images were resized to a standard dimension of 256^2^ pixels. There was no missing data. Augmentations included random horizontal flips and random affine transformations, such as translations, rotations, scaling, and shear transformations to enhance model robustness and generalization. For the binary classification task, we combined the moderate and severe classes, leaving a normal (14%) and an abnormal (86%) class. For the multi-class classification task, the class labels were normal (14%), moderate (36%), and severe (50%).

## AI Model and Training

We utilized a ResNet50 architecture, incorporating MC Dropout layers, which served as an approximation of Bayesian Neural Networks (BNNs) [18, 23, 24]. The final layers consisted of an MC Dropout layer, two linear transformations (512 units each), batch normalization, and ReLU activation, culminating in either softmax (multi-class) or sigmoid (binary) output layers. We used a learning rate of 0.001, the Adam optimizer, early stopping after 3 successive iterations, and ensured a stratified split across class labels to maintain the class label distribution across training, validation, and testing.

To mitigate the effects of class imbalance, we employed a weighted loss strategy during model training. The weighted loss function penalized the model based on the inverse of the class distribution. This weighted loss strategy ensured that the minority classes are given more weight in the learning process. This approach helped to counterbalance the skewed distribution of the dataset, allowing the model to better recognize and classify underrepresented classes.

## Active Learning Strategy

**Fig. 2:**
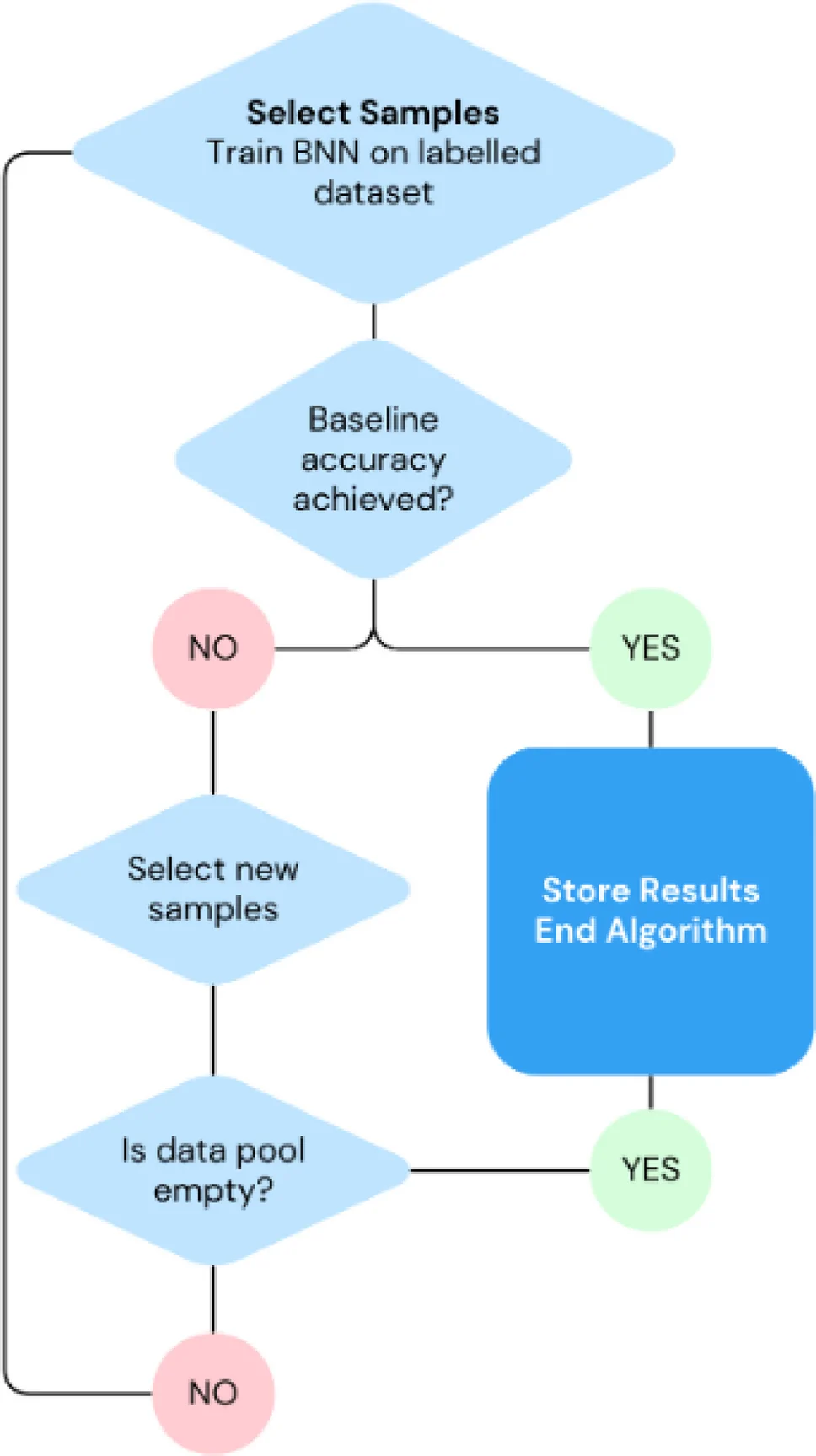
Overview of the active learning procedure. The process begins by selecting an initial batch of randomly chosen samples (25 per class severity) to train the Bayesian Neural Network (BNN). After training, we evaluate whether baseline accuracy has been achieved. If so, the result is stored, and the algorithm terminates. If not, a new batch of samples is selected based on the specified acquisition function (e.g., Entropy Sampling), and the BNN is retrained from scratch using the expanded labeled set. This iterative loop continues until either the data pool is exhausted, or baseline accuracy is reached

The active learning framework iteratively expanded the training set, *D*_train_ using acquisition functions to identify the most informative samples from an unlabeled pool, *D*_pool_. The process was randomly initialized with 25 labeled CXRs per severity class from *D*_train_, and in each subsequent iteration, 20 additional samples were acquired from *D*_pool_ using an acquisition function and labeled. This iterative sampling continued until either the model reached baseline diagnostic performance or the *D*_pool_ was exhausted. The active learning implementation logic can be seen in Fig. 2. For more details, see the first section in Online Resource.

The baselines used in both the binary and multi-class settings are BNN models trained on the entire training dataset, which represents 70% of the total dataset. The results on the testing set were used as a benchmark.

## Acquisition Function

Seven acquisition functions guided sample selection: Random Sampling, Entropy Sampling [25, 26], BatchBALD [27], Mean STD [28], Least Confidence [29], Margin Sampling [30], and Variation Ratios [31]. Detailed definitions and mathematical formulations are provided in the second section of the Online Resource.

## Evaluation Metrics

We evaluated the performance of the model using accuracy, negative log-likelihood (NLL), macro F1 score, precision, sensitivity, and specificity, area under the ROC curve (AU-ROC), and area under the precision-recall curve (AU-PRC). For simplicity, F1 score, precision, sensitivity, and specificity will imply their macro calculation, unless stated otherwise. Weighted and per-class metrics are provided in the Online Resources. Computational efficiency was evaluated by measuring Training Model Elapsed Time and Batch Acquisition Elapsed Time. Experiments were executed using Python on an RTX 2080 GPU, repeating each acquisition function evaluation with ten distinct random seeds. We showcase the result using the median with interquartile ranges (IQR) using the 25th and 75th percentiles.

We also compared the sampling techniques with Random Sampling. We used paired one-sided Wilcoxon signed-rank tests comparing each acquisition function to the random sampler at the 90%, 95%, and 99% significance levels [32]. The optimal acquisition function was identified based on the lowest median number of training data samples and the narrowest IQR across most evaluation metrics. For both classification tasks, we determined the percentage of training data at which this acquisition function first achieved baseline performance. This same subset of training data, selected by the optimal acquisition function, was subsequently used for Random Sampling as well. Evaluation metrics were computed using models trained on both the actively and randomly sampled subsets, and the mean and standard deviation were calculated.

The dashed line (-) in the result tables denotes that the active learning algorithm was terminated without achieving baseline performance. It is worth mentioning that if the random sampler did not reach the baseline metrics (represented by a dashed line), as it theoretically should have, it means that almost all of the training data were exhausted. If all of the data had been exhausted, the random sampler would have achieved baseline metric performance, since the same samples used in the baseline would have been used in the final iteration.

## Results

The data included 2199 CXRs from 963 patients, with the mean age of the patients being 59*.*2 ± 16*.*6 years and 49.9% (*n* = 481) of the patients being female. The average BMI was 32*.*7 ± 10*.*9. It is worth mentioning that 120 CXRs lacked demographic information and were omitted from Table 1. Of the labeled images, 1157 were severe, 836 mild, and 326 normal.

**Table 1:** Statistics and patient characteristics for the CXR datasets used in the study

Characteristic	Statistic
Number of images (N)	2199
Patients	963
Age (mean ± SD)	59.2 ± 16.6
BMI (mean ± SD)	32.7 ± 10.9
Female count	481 (49.9%)

## Binary Results

**Fig. 3:**
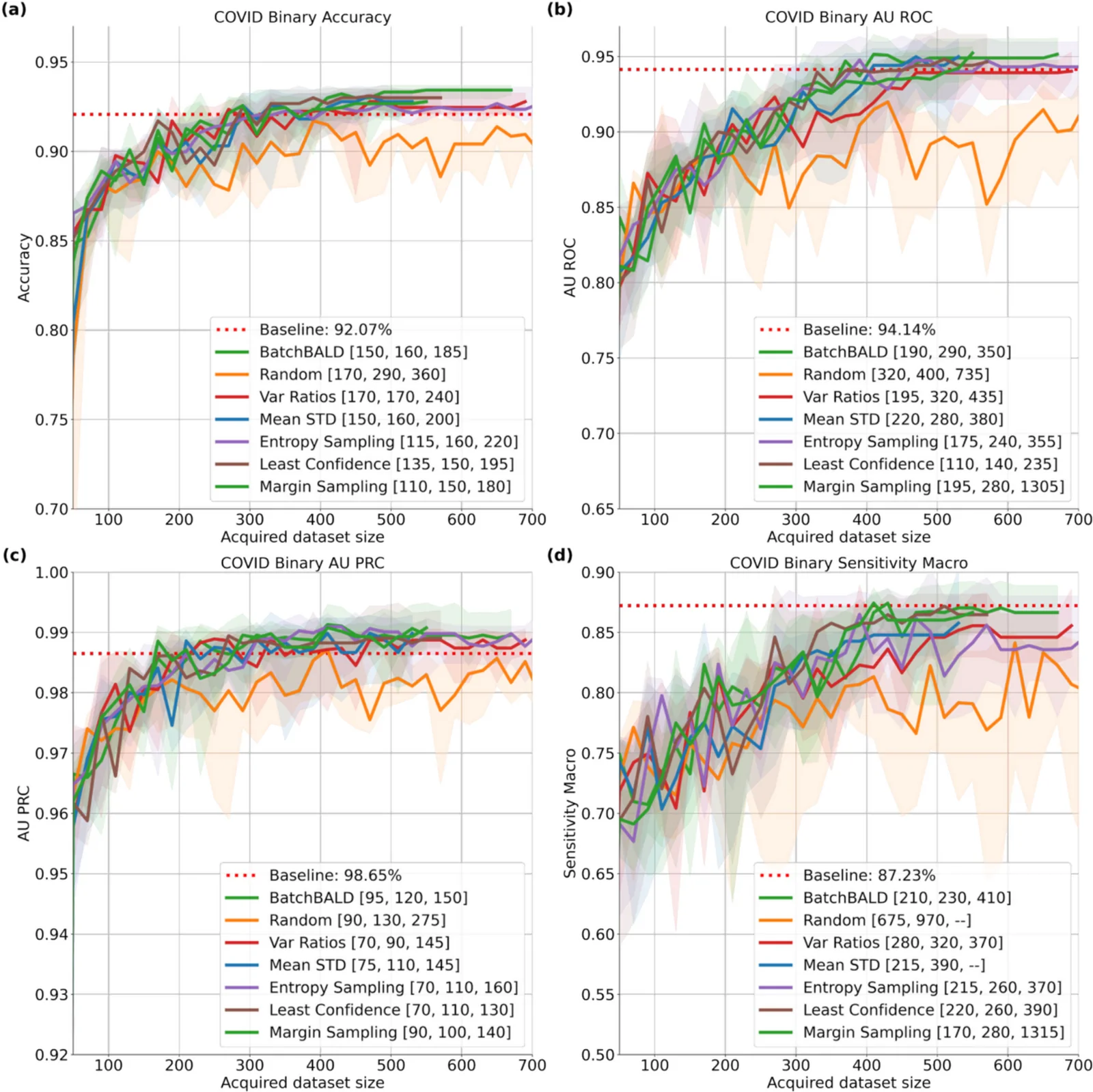
Binary classification results across various macro metrics as a function of the number of imaging data samples acquired: accuracy (**a**), AU-ROC (**b**), AU-PRC (**c**), sensitivity (**d**). The results are reported as [25th, 50th, 75th] percentiles. The baseline is denoted as a dotted line in red

The baseline results can be seen in Online Resource: Table 1. The results detailed in Fig. 3 were achieved using a baseline accuracy of 92.07% as a stopping criterion for the active learning algorithm. Table 2 shows a summary of the binary classification results. For all metrics, see Online Resource: Fig. 1.

**Table 2:** Binary classification results showing percentage of training data used until baseline performance is reached. The optimal median number of imaging data samples are represented in bold for each metric, and the corresponding acquisition function is deemed optimal

Acquisition function	Accuracy	AU-ROC	AU-PRC	Macro sensitivity	Macro F1	Macro specificity	Macro precision
Median (%)							
Random	17.87	24.65	8.01	59.77	57.30	59.77	15.40
BatchBALD	9.86**	17.87*	7.39	14.17**	21.57**	24.03*	11.09
Entropy Sampling	9.86*	14.79	6.78**	16.02***	24.65	16.02**	8.63*
Least Confidence	9.24**	8.63***	6.78	16.02***	**19.72**	16.02**	8.63***
Margin Sampling	9.24***	17.25	6.16*	17.25*	25.88	20.95	7.39***
Mean STD	9.86	17.25	6.78	24.03	24.65	-	8.63***
Var Ratios	10.47**	19.72	5.55*	19.72**	20.33**	25.88	10.47**
IQR (%)							
Random	[10.47, 22.18]	[19.72, 45.29]	[5.55, 16.94]	[41.59, -]	[38.82, -]	[39.74, -]	[9.55, 19.72]
BatchBALD	[9.24, 11.40]**	[11.71, 21.57]*	[5.85, 9.24]	[12.94, 25.26]**	[13.56, 28.03]**	[12.94, 82.24]*	[7.09, 15.71]
Entropy Sampling	[7.09, 13.56]*	[10.78, 21.87]	[4.31, 9.86]**	[13.25, 22.80]***	[15.71, -]	[14.48, 38.51]**	[6.78, 11.71]*
Least Confidence	[8.32, 12.01]**	[6.78, 14.48]***	[4.31, 8.01]	[13.56, 24.03]***	**[16.64, -]**	[13.56, 23.41]**	[6.78, 10.17]***
Margin Sampling	[6.78, 11.09]***	[12.01, 80.39]	[5.55, 8.63]	[10.47, 81.01]*	[13.86, -]	[11.09, 83.16]	[4.62, 9.24]***
Mean STD	[9.24, 12.32]	[13.56, 23.41]	[4.62, 8.93]	[13.25, -]	[6.16, 82.86]	[23.41, -]	[5.85, 10.47]***
Var Ratios	[10.47, 14.79]**	[12.01, 26.80]	[4.31, 8.93]*	[17.25, 22.80]**	[12.01, 22.80]**	[17.25, 85.32]	[8.63, 12.32]**

The interquartile range (IQR) column represents the 25th and 75th percentiles. The dashed line (-) denotes that the active learning algorithm was terminated without achieving baseline performance. The stars denote statistical significance compared to the random baseline: ^*^p < 0*.*10, ^**^p < 0*.*05, ^***^p < 0*.*01. The values in bold represent the lowest median values and their corresponding IQRs

Our findings indicate that Least Confidence performs the best in the binary setting, achieving baseline accuracy of around 93% with only 9.24% (IQR: [8.32*,* 12.01], *p* = 0.061) of the training data outperforming more complex acquisition functions like BatchBALD and Variation Ratios, which required 9.86% (IQR: [9.24*,* 11.40], *p* = 0.021) and 10.47% (IQR: [10.47, 14.79], *p* = 0.016) of the training data, respectively. Furthermore, our approach surpasses the baseline performance for AU-ROC and AU-PRC with less training data. In the binary setting, Least Confidence achieves an AU-ROC of 95% and an AU-PRC of 99%, surpassing the baseline AU-ROC of 94% while matching the AU-PRC baseline, using only 8.63% (IQR: [6.78*,* 14.48], *p* < 0.01) of the training data for AU-ROC and only 6.78% (IQR: [4.31*,* 8.01], *p* = 0.109) of the training data for AU-PRC. It is worth mentioning that Entropy Sampling and Margin Sampling achieved baseline AU-PRC with similar data points (6.78%, *p* = 0.031 and 6.16%, *p* = 0.091). Of note, all acquisition functions outperform Random Sampling, except for Mean STD under specificity.

## Multi-class Results

**Fig. 4:**
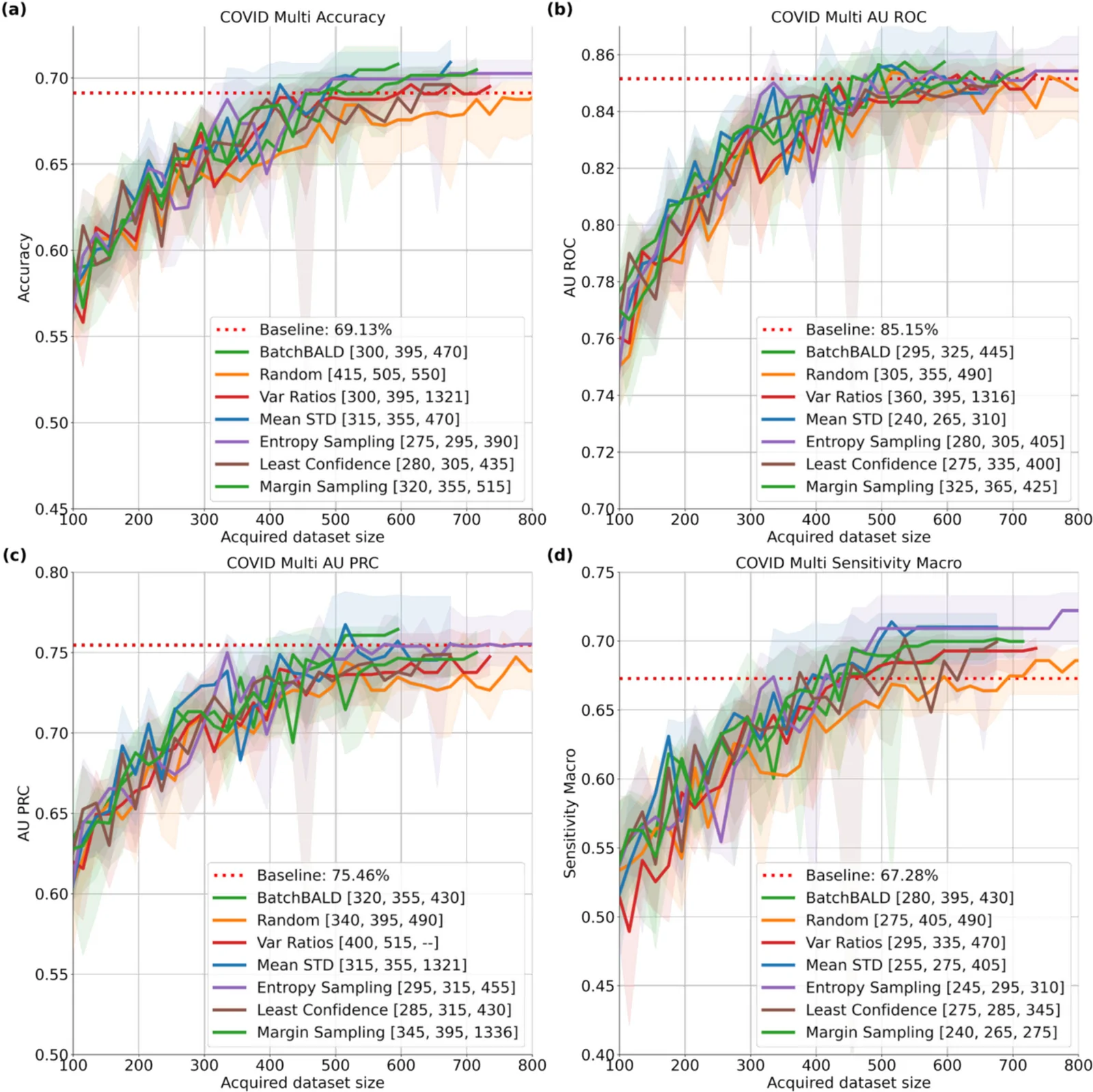
Multi-class classification results across various macro metrics as a function of the number of imaging data samples acquired: accuracy (**a**), AU-ROC (**b**), AU-PRC (**c**), sensitivity (**d**). The results are reported as [25th, 50th, 75th] percentiles. The baseline is denoted as a dotted line in red

The baseline results can be seen in Online Resource: Table 2. After using a baseline accuracy of 69.13% as a stopping criterion for the active learning algorithm, we achieved the following results as seen in Fig. 4. Further, Table 3 shows a summary of the multi-class classification results. For all metrics, see Online Resource: Fig. 2.

**Table 3:** Multi-class classification results showing percentage of training data used until baseline performance is reached. The optimal median number of imaging data samples are represented in bold for each metric, and the corresponding acquisition function is deemed optimal

Acquisition function	Accuracy	AU-ROC	AU-PRC	Macro sensitivity	Macro F1	Macro specificity	Macro precision
Median (%)							
Random	32.35	21.87	29.88	24.95	19.41	29.88	16.33
BatchBALD	24.34	21.87	**21.87**	24.34	17.56	24.34	**12.01**
Entropy Sampling	18.79*	23.11	28.03	18.18**	17.56	24.34**	**12.01**
Least Confidence	20.64*	20.64	24.34	17.56*	15.71**	24.95	14.48
Margin Sampling	23.11	23.72	25.57	**16.94**	17.56	20.64**	14.48
Mean STD	21.87*	**18.79**	**21.87**	18.79*	19.41	26.80	12.63
Var Ratios	24.95	24.34	31.73	20.64	23.11	32.96	14.48
IQR (%)							
Random	[25.57, 42.51]	[18.79, 30.19]	[23.11, 33.58]	[16.94, 30.19]	[16.02, 23.72]	[23.41, 86.01]	[9.24, 19.10]
BatchBALD	[18.48, 28.96]	[19.72, 28.34]	**[19.72, 26.49]**	[17.25, 26.49]	[16.02, 21.26]	[20.33, 26.80]	**[10.78, 14.17]**
Entropy Sampling	[17.25, 28.65]*	[17.56, 27.73]	[21.26, 83.55]	[17.25, 19.41]**	[13.86, 24.03]	[18.18, 32.96]**	**[9.55, 13.25]**
Least Confidence	[18.18, 33.58]*	[16.94, 26.49]	[19.41, 31.73]	[16.94, 21.26]*	[13.56, 17.87]**	[18.48, 31.73]	[11.09, 15.40]
Margin Sampling	[20.02, 40.67]	[20.02, 27.73]	[23.41, 82.32]	**[14.79, 17.87]**	[14.79, 21.26]	[16.94, 29.88]**	[13.56, 17.87]
Mean STD	[19.41, 28.96]*	**[16.02, 81.08]**	**[19.41, 81.39]**	[15.71, 24.95]*	[16.33, 24.34]	[19.41, 32.66]	[12.01, 16.33]
Var Ratios	[18.48, 84.17]	[23.41, 100.00]	[24.65, 100.00]	[18.18, 28.96]	[17.56, 25.26]	[23.41, 86.01]	[13.25, 15.71]

The interquartile range (IQR) column represents the 25th and 75th percentiles. The dashed line (-) denotes that the active learning algorithm was terminated without achieving baseline performance. The stars denote statistical significance compared to the random baseline: ^*^p < 0*.*10, ^**^p < 0*.*05, ^***^p < 0*.*01. The values in bold represent the lowest median values and their corresponding IQRs

Our findings indicate that Mean STD performs the best in a multi-class setting, achieving a baseline accuracy of 69% with only 21.87% (IQR: [19*.*41*,* 28.96], *p* = 0.076) of the training data. In the multi-class setting, Mean STD achieves an AU-ROC of 85% and an AU-PRC of 75%, matching the baseline AU-ROC of 85% and AU-PRC of 75%, respectively, using only 18.79% (IQR: [16.02*,* 81.08], *p* = 0.606) and 21.87% (IQR: [19.41*,* 81.39], *p* = 0.445) of the training data, respectively. This sampling technique outperforms more complex acquisition functions like BatchBALD and Variation Ratios. BatchBALD required 24.34% (IQR: [18.48*,* 28.96], *p* = 0.116); 21.87% (IQR: [19.72*,* 28.34], *p* = 0.510); and 21.87% (IQR: [19*.*72*,* 26.49], *p* = 0.144) of the training data to achieve baseline performance for accuracy, AU-ROC, and AU-PRC, respectively. Variation Ratios required 24.95% (IQR: [18.48*,* 84.17], *p* = 0.455); 24.34% (IQR: [23.41*, −*], *p* = 0.936); and 31.73% (IQR: [24*.*65*, −*], *p* = 0.746) of the training data to achieve baseline performance for accuracy, AU-ROC, and AU-PRC, respectively. It is worth mentioning that Entropy Sampling achieved baseline accuracy using less data albeit a slightly wider IQR, 18.79% (IQR: [17.25*,* 28.65], *p* = 0.094). Of note, all acquisition functions outperform Random Sampling, except Variational Ratios under AU-ROC, AU-PRC, specificity, and F1.

## Comparing Active Sampler with Random Sampling

**Fig. 5:**
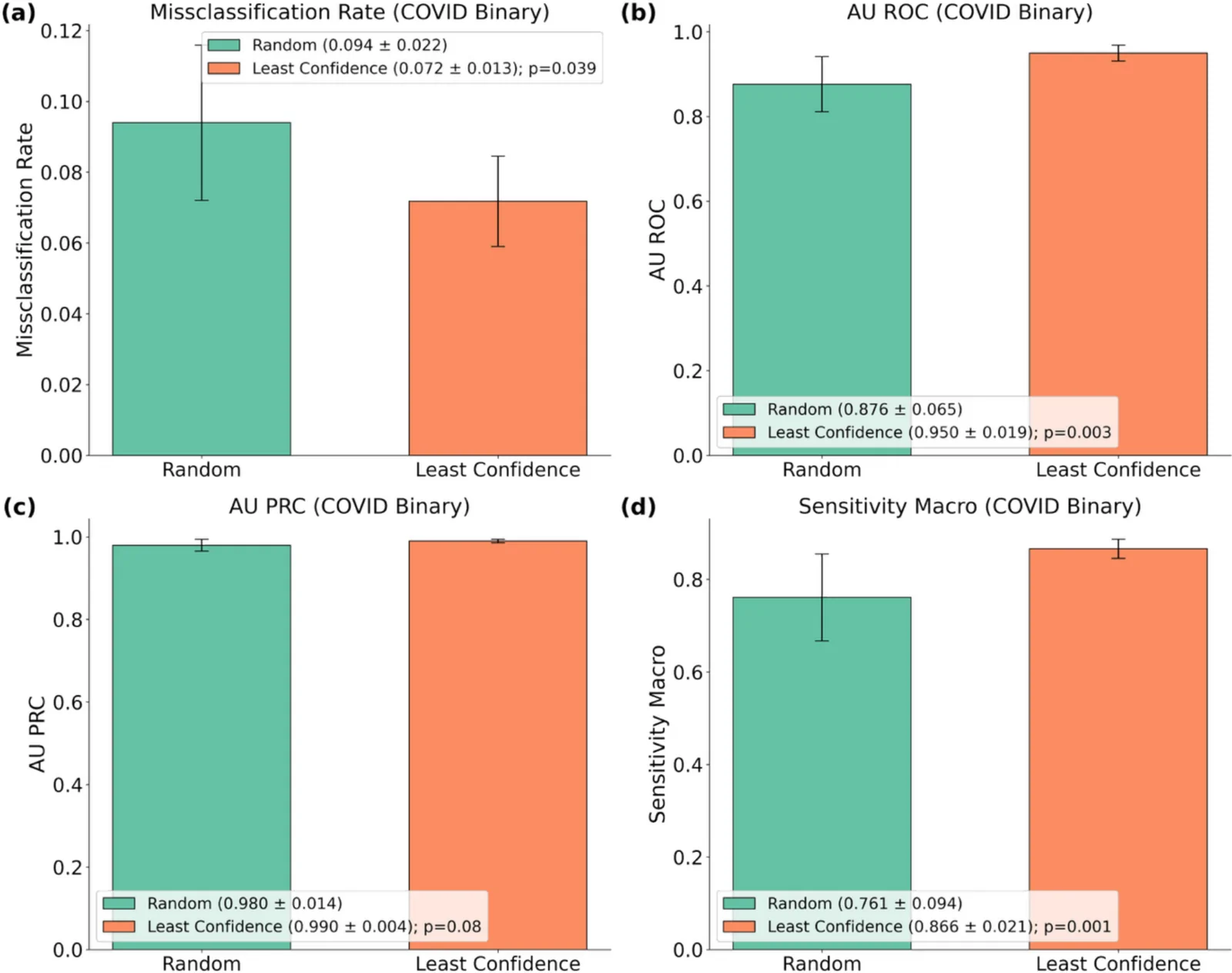
Binary classification error comparison between Least Confidence and Random Sampling using only 9.24% of the imaging data samples across various metrics: misclassification rate (**a**), AU-ROC (**b**), AU-PRC (**c**), sensitivity (**d**)

**Fig. 6:**
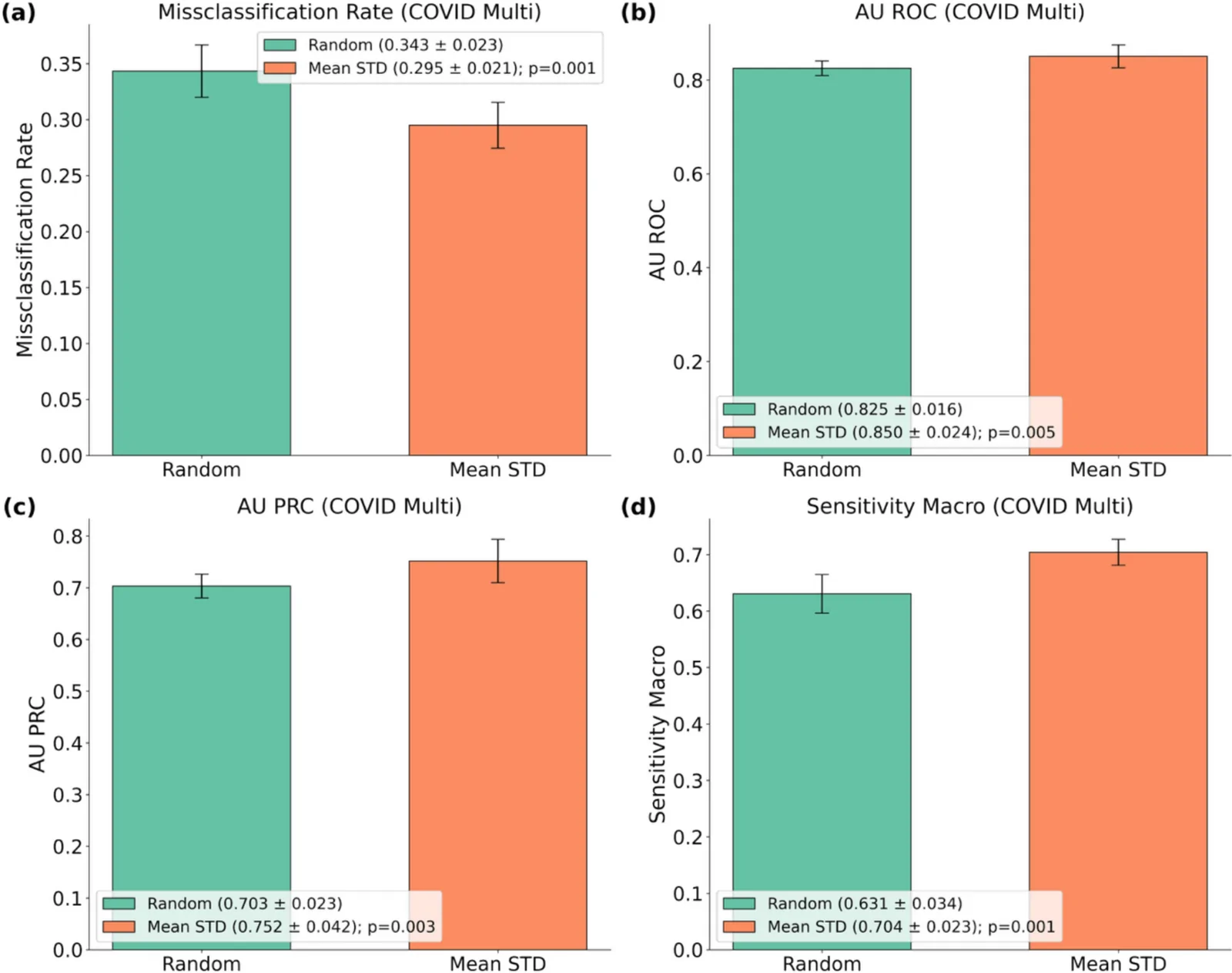
Multi-class classification error comparison between Mean STD and Random Sampling using only 21.87% of the imaging data samples across various metrics: misclassification rate (**a**), AU-ROC (**b**), AU-PRC (**c**), sensitivity (**d**)

As seen in Figs. 5 and 6, relative to all metrics, Least Confidence (binary) and Mean STD (multi) outperform Random Sampling using the same subset of data, while still achieving and sometimes surpassing baseline level performance with minimal data used. The misclassification error of the random sampler is higher than that of Least Confidence and Mean STD (0*.*094 ± 0*.*022 vs. 0*.*072 ± 0*.*013 for Lesat Confidence, *p* = 0.039, and 0*.*343 ± 0*.*023 vs. 0*.*295 ± 0*.*022 for Mean STD, *p* < 0.01), demonstrating the powerful results and consistency of the samplers. For all metrics, see Online Resource: Figs. 3 and 4.

## Sample Distribution and Computational Efficiency Across Acquisition Functions

As shown in Online Resource: Table 3 and Fig. 5, Random Sampling largely reflected the original class distribution of the dataset, 13.3% normal and 86.7% abnormal in the binary setting and 13.7%, 37.0%, and 49.3% for normal, moderate, and severe in the multi-class setting. In contrast, Least Confidence and Mean STD exhibited a shift toward oversampling the minority class. Least Confidence selected 35.7% normal samples in the binary setting, while Mean STD sampled 30.5% in the multi-class setting, one of the highest among all samplers. In multi-class setting, Mean STD sampled 30.5% normal, 41.8% moderate, and 27.8% severe, shifting focus toward the moderate class and sampling fewer from the overrepresented severe class. All acquisition functions besides Random Sampling followed a similar trend, sampling more from the moderate and normal classes and less from severe.

Timing analysis (Online Resource Table 4 and Fig. 6) showed that batch acquisition time was negligible for Random Sampling and highest for BatchBALD, averaging 60–70 s per iteration. Least Confidence and Mean STD maintained relatively low acquisition times (43–46 s) while still achieving strong performance. Training times remained broadly comparable across all acquisition functions (245–309 s), with Mean STD trending slightly faster on average.

**Table 4:** Optimal acquisition function based on desired metric

Metric	Binary	Multi
Accuracy	Least Confidence^∗^	Entropy Sampling
AU-ROC	Least Confidence	Mean STD
AU-PRC	Variational Ratios	BatchBALD^∗∗∗^
Sensitivity	BatchBALD	Margin Sampling
F1	Least Confidence	Least Confidence
Specificity	Least Confidence^∗∗^	Margin Sampling
Precision	Margin Sampling	Entropy Sampling^∗∗∗∗^

^∗^Margin Sampling has the same median number of samples but a larger IQR across trials

^∗∗^Entropy Sampling has the same median number of samples but a larger IQR across trials

^∗∗∗^Mean STD has the same median number of samples but a larger IQR across trials

^∗∗∗∗^BatchBALD has the same median number of samples but a larger IQR across trials

## Optimizing for Each Metric

A summary of the optimal acquisition function based on each desired metric is shown in Table 4. We can see that Least Confidence is optimal if, with minimum data, the use case is to maximize accuracy, AU-ROC, F1, and specificity in the binary setting and AU-PRC, F1, and specificity in the multi-class setting. Mean STD, however, can be used to maximize AU-ROC and AU-PRC in the multi-class setting. Other sampling techniques, like Entropy Sampling and Margin Sampling, are also useful for maximizing specificity in the binary and multi-class setting, respectively.

## Discussion

This study demonstrates that deep active learning can substantially reduce labeling requirements for COVID-19 CXR classification, particularly when using Least Confidence for binary and Mean STD for multi-class tasks. These acquisition functions achieved baseline diagnostic accuracy with only 9.24% and 21.87% of the training data. Outperforming more complex methods like BatchBALD and Variation Ratios, these methods reduced labeling needs by up to 48% and 32%, respectively.

Least Confidence and Mean STD also exhibited narrower IQRs for most metrics indicating consistent and reproducible performance. This is critical in clinical deployment where variability can undermine trust in AI. Additionally, their low acquisition latency further supports their practicality over methods like BatchBALD, which are computationally expensive and less consistent. These findings show the practicality of combining simpler acquisition functions with a weighted loss strategy to manage class imbalance, a common issue in medical imaging datasets [12, 13, 17, 33–36].

Previous work on label efficiency in radiology has often relied on more complex approaches. Wu et al. [16] achieved 86.6% accuracy with 42% labeled CTs using a loss prediction module and a balanced severity cohort. Mahapatra et al. [37]. reached 95.3% AUROC using 35% of labeled CXRs with a combination of BNNs and generative adversarial networks. Nazir et al. [17] applied discriminative clustering to improve sampling under class imbalance. In contrast, our method achieved comparable or better performance with just 9.24–21.87% of labeled data, using standard acquisition functions and architectures, making it more scalable and easier to deploy in clinical settings.

Our approach is also flexible and adaptable in real-world settings, allowing different acquisition functions to be chosen based on clinical priorities. For example, Least Confidence is preferable for F1 focused multi-class tasks, whereas Margin Sampling is preferred in binary settings when precision is critical. Such adaptability is vital in scenarios like pandemics or rare disease contexts, where rapid deployment of diagnostic models is essential and labeling resources are scarce [38, 39].

There are several limitations in our study. First, because multiple images can originate from the same patient, we employed an imbalance-aware patient-stratified data split to prevent leakage. However, future work should explore patient-aware active learning strategies that explicitly model within-patient correlation during sampling but under an imbalanced setting. Second, our dataset comprises COVID-19 CXRs from a single institution, which limits generalizability. We used this cohort as a case study for lung-disease classification due to its high-quality annotations, but validating the proposed framework on non-COVID and multi-center datasets remains an essential next step to confirm robustness across institutions and disease types. Third, this work focused on active learning acquisition functions under class imbalance. Extending the analysis to include semi-supervised, self-supervised, or class-rebalancing techniques, such as over- and under-sampling or adaptive augmentation, would be valuable future directions. In this study, we mitigated imbalance through weighted-loss optimization, ensuring that minority classes contributed proportionally to model learning. Finally, investigating patterns in selected images based on different acquisition functions may provide valuable insights into epistemic and aleatoric uncertainties, assisting radiologists with complex cases.

Overall, our analysis of sampling patterns shows the operational viability of Least Confidence and Mean STD. Their tendency to oversample ambiguous or underrepresented classes mitigates class imbalance during learning. Overall, this simple yet effective approach reduces annotation burden and training cost without sacrificing performance, making it well-suited for scalable radiology AI workflows beyond COVID-19 and CXRs.

## Conclusion

Deep active learning integrated with a BNN using MC dropout and weighted loss achieves competitive diagnostic accuracy on imbalanced CXR datasets using only 9.24% of the training data for binary classification and 21.87% for multi-class classification. This significantly reduced the amount of data needed to train AI diagnostic models and radiologists’ data labeling requirements. Among seven sampling techniques from naive techniques like Random Sampling to advanced and complicated techniques like BatchBALD, Least Confidence and Mean STD emerged as the most effective techniques, outperforming more complex and computationally expensive acquisition methods by selectively targeting the most informative samples in imbalanced datasets. This study offers the opportunity to decrease the cost and amount of data annotation requirement, accelerating the deployment of AI-driven diagnostic tools. It supports radiologists in labeling the least amount of data needed to achieve the highest performance metric they desire, such as AU-ROC, sensitivity, and/or specificity.

## Supplementary Information

Below is the link to the electronic supplementary material.

ESM1 (DOCX.4.77 MB)

## Data Availability

The datasets employed in this study are available under reasonable request. Please contact Dr. Carlo De Cecco and Dr. Ali Adibi for any request regarding the data.
