# Supplementary material for: Deep Active Learning for Lung Disease Severity Classification from Chest X-rays: Learning with Less Data in the Presence of Class Imbalance
Source: J Imaging Inform Med. 2025 Dec 10;39(5):3930–41. doi: 10.1007/s10278-025-01752-8 (PMC13694490; doi:10.1007/s10278-025-01752-8)
Supplement: Supplementary file 1 [file 10278_2025_1752_MOESM1_ESM.docx]

Deep Active Learning for Lung Disease Severity Classification from Chest X-rays: Learning with Less Data in the Presence of Class Imbalance

**Journal Name:** Journal of Imaging Informatics

**Authors:** Roy M. Gabriel, Mohammadreza Zandehshahvar, Marly van Assen, Nattakorn Kittisut, Kyle Peters, Carlo N. De Cecco, Ali Adibi

**Corresponding Author**

Ali Adibi, PhD

School of Electrical and Computer Engineering

Georgia Institute of Technology

791 Atlantic Dr NW

Atlanta, GA 30332, USA

Email: ali.adibi@ece.gatech.edu

Phone: 404-385-2738

Office: Bunger Henry 105

# Supplementary Material Overview

The supplementary material contains additional implementation details, acquisition function definitions, baseline tables, extended evaluation plots, and sampling/training time analysis not included in the main manuscript.

# 1. Active Learning Implementation

The following definitions clarify the framework and notation used in this paper:

- **Training Pool (**$D_{pool}$**):** This is the set of all unlabeled data points available for potential labeling. Formally, let $D_{pool}=\left\{ x_{1}, x_{2}, \ldots, x_{m} \right\}$*,* where $x_{i}$ represents an individual data point in the pool.
- **Training Set (**$D_{train}$**):** This is the set of labeled data points used to train the model. Initially, $D_{train}$ is populated with a predetermined number of labeled data points from each class. As active learning progresses, $D_{train}$ is updated with new labeled samples.
- **Acquisition Function:** An acquisition function evaluates the informativeness of each data point in $D_{pool}$ to determine which points should be labeled next. For each acquisition function, the input is denoted by the vector $\boldsymbol{x}$, where $\boldsymbol{x}\boldsymbol{\in}D_{pool}$.
- **Selected Samples (***S_t_***):** At each iteration *t*, an acquisition function selects a subset $S_{t} \subset D_{pool}$ of $n_{t}$ samples to be labeled. The size of $S_{t}$ and the value of $n_{t}$ is typically determined by the active learning strategy or a predefined budget.
- **Updated Training Set (**$D_{train}^{t}$ **)***:* After labeling the selected samples, the training set is updated to $D_{\mathrm{train}}^{t}= D_{\mathrm{train}}^{t-1} {\cup S}_{t}$,where $D_{\mathrm{train}}^{t-1}$ is the training set before iteration t.

The BNN was trained with the following specifics:

- **Learning Rate:** A fixed learning rate of 0*.*001 is used throughout the training.
- **Optimizer:** The Adam optimizer is utilized for its adaptive learning rate properties.
- **MC Dropout:** Applied during training and inference to approximate Bayesian uncertainty.
- **Early Stopping:** Training stops when no improvement is seen over three successive iterations.
- **Initial Stratified Split:** Ensures the label distribution is consistent across all random seeds and initializations. This means that the distribution of class labels is uniform across different splits generated by the same random seed but may vary between different seeds. This approach maintains the inherent nature of the data distribution across different experiments.

# 2. Acquisition Functions

2.1. *Random Sampling*

Random Sampling selects data points uniformly at random from the unlabeled pool, $D_{pool}$. This method does not leverage any information from the model or data and serves as a baseline for comparing the effectiveness of more sophisticated acquisition functions.

2.2. *Entropy Sampling*

Entropy Sampling selects samples that maximize the entropy of the predictive distribution. Entropy, defined as:

$$H\left( \boldsymbol{x} \right)= - \sum_{c=1}^{C} \hat{p}\left( y=c | \boldsymbol{x} \right)\log\hat{p}\left( y=c \right| \boldsymbol{x})$$

is a measure of uncertainty, and this method chooses instances where the model’s predictive distribution is most uncertain. The predictive probability, denoted as  $\hat{p}\left( y=c | \boldsymbol{x} \right)$, represents the BNN model’s estimated probability that a data point **x** belongs to class *c*.

2.3. *Batch BALD*

BatchBALD extends the BALD method to batch mode active learning, where it jointly maximizes the mutual information between the model predictions and the model parameters across multiple points. This helps in selecting diverse and informative batches of data points.

The mutual information $I$ for a batch $\boldsymbol{b}$ is defined as:

$$I\left( \boldsymbol{b;\theta} | D \right)= H\left( \boldsymbol{b} | D \right)- \mathbb{E}_{\boldsymbol{\theta}\sim p\left( \boldsymbol{\theta} | D \right)}\left[ H\left( \boldsymbol{b} | \boldsymbol{\theta}, D \right) \right]$$

where *H* is the entropy, $\boldsymbol{\theta}$ are the model parameters, and $D$ is the dataset.

2.4. *Mean STD*

Mean STD selects samples based on the standard deviation of the predictive probabilities. It aims to choose instances where the model is most uncertain, as indicated by a high standard deviation in the predicted class probabilities. This method is effective in highlighting regions of the input space where the model’s predictions are less certain.

The standard deviation for a data point **x** is given by:

$$STD\left( \boldsymbol{x} \right)=\sqrt{\frac{1}{C}\sum_{c=1}^{C} \left( \hat{p}\left( y=c | \boldsymbol{x} \right)-\bar{\hat{p}\left( y=c | \boldsymbol{x} \right)} \right)^{2}}$$

where $C$ is the number of classes and $\hat{p}\left( y=c | \boldsymbol{x} \right)$ is the mean predictive probability. In our approach, the mean predictive probability is calculated by averaging the predicted probabilities over multiple stochastic forward passes using MC Dropout to account for model uncertainty.

2.5. *Least Confidence*

Least Confidence selects samples where the model has the lowest confidence in its predictions. The confidence is typically defined as the highest class probability predicted by the model. This method targets instances where the model’s most confident prediction is still relatively low.

The confidence score for a data point $\boldsymbol{x}$ is given by:

$$LC\left( \boldsymbol{x} \right)=1-\max_{c} \hat{p}\left( y=c | \boldsymbol{x} \right)$$

where $\hat{p}\left( y=c | \boldsymbol{x} \right)$ is the predicted probability for class $c$.

2.6. *Margin Sampling*

Margin Sampling selects instances based on the difference between the highest and second highest predicted probabilities. The margin is given by:

$$M\left( \boldsymbol{x} \right) =\hat{p}\left( \hat{y}=c_{1} | \boldsymbol{x} \right) -\hat{p}\left( \hat{y}=c_{2} | \boldsymbol{x} \right)$$

where $\hat{p}\left( \hat{y}=c_{1} | \boldsymbol{x} \right)$ and $\hat{p}\left( \hat{y}=c_{1} | \boldsymbol{x} \right)$ are the first and second highest predicted class

probabilities, respectively. Smaller margins indicate higher uncertainty and thus are selected.

2.7. *Variation Ratios*

Variation Ratios is an uncertainty-based acquisition function that selects instances based on the variability in predictions across multiple model iterations. It is defined as:

$$VR\left( \boldsymbol{x} \right)=1-\frac{1}{T}\sum_{t=1}^{T} \mathbb{I}\left[ \hat{y}^{t}=\hat{y}^{*} \right]$$

where $\hat{y}^{t}$ is the predicted class label for the data point $\boldsymbol{x}$ during the $t$^th^ forward pass, $\hat{y}^{*}$ is the mode of the predicted class label over the $T$ stochastic Monte Carlo forward passes, and $\mathbb{I}\left[ \hat{y}^{t}=\hat{y}^{*} \right]$ is an indicator function that equals one if the prediction in the $t$^th^ pass is equal to the most frequent prediction $\hat{y}^{*}$, and zero otherwise. This method captures the model’s predictive uncertainty by considering the variability in class predictions.

# 3. Tables and Figures

Table 1: Baseline Performance Metrics on Binary Dataset

| **Metric** | **Value** |
| --- | --- |
| Accuracy | 0.9207 |
| NLL | 0.2379 |
| F1 Macro | 0.8607 |
| F1 Weighted | 0.9219 |
| Precision Macro | 0.8503 |
| Precision Weighted | 0.9236 |
| Sensitivity Macro | 0.8723 |
| Sensitivity Weighted | 0.9207 |
| Specificity Macro | 0.8723 |
| Specificity Weighted | 0.8239 |
| AU-ROC | 0.9414 |
| AU-PRC | 0.9865 |
| F1 Class 0 | 0.7692 |
| F1 Class 1 | 0.9521 |
| Precision Class 0 | 0.7407 |
| Precision Class 1 | 0.9598 |
| Sensitivity Class 0 | 0.8000 |
| Sensitivity Class 1 | 0.9446 |
| Specificity Class 0 | 0.9446 |
| Specificity Class 1 | 0.8000 |

Table 2: Baseline Performance Metrics on Multi Dataset

| **Metric** | **Value** |
| --- | --- |
| Accuracy | 0.6913 |
| NLL | 0.5681 |
| F1 Macro | 0.6684 |
| F1 Weighted | 0.6823 |
| Precision Macro | 0.6815 |
| Precision Weighted | 0.6883 |
| Sensitivity Macro | 0.6728 |
| Sensitivity Weighted | 0.6913 |
| Specificity Macro | 0.8232 |
| Specificity Weighted | 0.7782 |
| AU-ROC | 0.8515 |
| AU-PRC | 0.7546 |
| F1 Class 0 | 0.6997 |
| F1 Class 1 | 0.5240 |
| F1 Class 2 | 0.7815 |
| Precision Class 0 | 0.7110 |
| Precision Class 1 | 0.5918 |
| Precision Class 2 | 0.7416 |
| Sensitivity Class 0 | 0.7139 |
| Sensitivity Class 1 | 0.4755 |
| Sensitivity Class 2 | 0.8291 |
| Specificity Class 0 | 0.9408 |
| Specificity Class 1 | 0.8328 |
| Specificity Class 2 | 0.6959 |

Table 3: Class distribution (% of selected samples) reported as mean ± standard deviation for each acquisition function across binary and multi-class classification tasks. Corresponds to Figure 5.

| **Acquisition Function** | **Binary: Normal / COVID** | **Multi: Normal / Moderate / Severe** |
| --- | --- | --- |
| BatchBALD | 32*.*2 *±* 21*.*6 / 67*.*8 *±* 21*.*6 | 30*.*8 *±* 18*.*1 / 41*.*4 *±* 14*.*1 / 27*.*9 *±* 15*.*9 |
| Entropy Sampling | 35*.*3 *±* 20*.*3 / 64*.*7 *±* 20*.*3 | 21*.*6 *±* 15*.*8 / 46*.*9 *±* 12*.*6 / 31*.*5 *±* 16*.*0 |
| Least Confidence | 35*.*7 *±* 20*.*6 / 64*.*3 *±* 20*.*6 | 22*.*1 *±* 14*.*9 / 46*.*9 *±* 13*.*0 / 31*.*0 *±* 15*.*0 |
| Margin Sampling | 36*.*5 *±* 21*.*0 / 63*.*5 *±* 21*.*0 | 17*.*7 *±* 10*.*2 / 47*.*0 *±* 11*.*5 / 35*.*3 *±* 12*.*5 |
| Mean STD | 34*.*4 *±* 22*.*1 / 65*.*6 *±* 22*.*1 | 30*.*5 *±* 20*.*9 / 41*.*8 *±* 15*.*7 / 27*.*8 *±* 18*.*2 |
| Random | 13*.*3 *±* 7*.*4 / 86*.*7 *±* 7*.*4 | 13*.*7 *±* 7*.*6 / 37*.*0 *±* 10*.*7 / 49*.*3 *±* 10*.*6 |
| Var Ratios | 35*.*0 *±* 20*.*2 / 65*.*0 *±* 20*.*2 | 22*.*7 *±* 15*.*3 / 47*.*2 *±* 13*.*5 / 30*.*1 *±* 14*.*5 |

Table 4: Training and acquisition (batch) time per iteration (in seconds), reported as mean ± standard deviation across binary and multi-class settings. Corresponds to Figure 6.

| **Acquisition Function** | **Binary: Train / Batch Time (s)** | **Multi: Train / Batch Time (s)** |
| --- | --- | --- |
| BatchBALD | 270*.*0 *±* 119*.*7 / 60*.*6 *±* 9*.*6 | 266*.*5 *±* 102*.*7 / 69*.*5 *±* 11*.*1 |
| Entropy Sampling | 263*.*0 *±* 112*.*8 / 44*.*3 *±* 7*.*0 | 285*.*8 *±* 129*.*6 / 41*.*3 *±* 8*.*2 |
| Least Confidence | 263*.*1 *±* 111*.*9 / 46*.*4 *±* 6*.*8 | 308*.*7 *±* 126*.*8 / 43*.*5 *±* 5*.*6 |
| Margin Sampling | 279*.*4 *±* 126*.*8 / 46*.*4 *±* 5*.*8 | 279*.*2 *±* 115*.*6 / 42*.*4 *±* 7*.*0 |
| Mean STD | 245*.*4 *±* 102*.*4 / 46*.*3 *±* 5*.*8 | 271*.*9 *±* 132*.*0 / 43*.*7 *±* 6*.*4 |
| Random | 253*.*9 *±* 109*.*9 / 0*.*0 *±* 0*.*0 | 271*.*7 *±* 120*.*5 / 0*.*0 *±* 0*.*0 |
| Var Ratios | 277*.*8 *±* 121*.*8 / 45*.*0 *±* 6*.*3 | 287*.*8 *±* 126*.*6 / 43*.*7 *±* 6*.*1 |

Table 5: COVID Binary: Macro metrics (mean ± std across seeds) when active learning algorithm terminated.

| **Acquisition Function** | **Accuracy** | **Macro F1** | **Macro Precision** | **Macro Sensitivity** | **Macro Specificity** | **AU-ROC** | **AU-PRC** |
| --- | --- | --- | --- | --- | --- | --- | --- |
| **Random** | 0.903 ± 0.042 | 0.819 ± 0.056 | 0.812 ± 0.075 | 0.846 ± 0.042 | 0.846 ± 0.042 | 0.935 ± 0.029 | 0.987 ± 0.007 |
| **Entropy Sampling** | 0.928 ± 0.012 | 0.848 ± 0.031 | 0.855 ± 0.028 | 0.849 ± 0.055 | 0.849 ± 0.055 | 0.944 ± 0.023 | 0.988 ± 0.007 |
| **Least Confidence** | 0.928 ± 0.013 | 0.856 ± 0.024 | 0.848 ± 0.033 | 0.866 ± 0.022 | 0.866 ± 0.022 | 0.950 ± 0.020 | 0.990 ± 0.005 |
| **Mean STD** | 0.924 ± 0.016 | 0.847 ± 0.030 | 0.843 ± 0.038 | 0.863 ± 0.051 | 0.863 ± 0.051 | 0.937 ± 0.039 | 0.985 ± 0.012 |
| **Margin Sampling** | 0.927 ± 0.005 | 0.850 ± 0.016 | 0.848 ± 0.017 | 0.857 ± 0.034 | 0.857 ± 0.034 | 0.946 ± 0.015 | 0.989 ± 0.004 |
| **BatchBALD** | 0.933 ± 0.007 | 0.860 ± 0.018 | 0.862 ± 0.018 | 0.862 ± 0.037 | 0.862 ± 0.037 | 0.950 ± 0.018 | 0.989 ± 0.006 |
| **Variational Ratios** | 0.927 ± 0.010 | 0.850 ± 0.020 | 0.848 ± 0.023 | 0.858 ± 0.039 | 0.858 ± 0.039 | 0.939 ± 0.025 | 0.987 ± 0.008 |

Table 6: COVID Binary: Weighted metrics (mean ± std across seeds) when active learning algorithm terminated.

| **Acquisition Function** | **Weighted F1** | **Weighted Precision** | **Weighted Sensitivity** | **Weighted Specificity** |
| --- | --- | --- | --- | --- |
| **Random** | 0.907 ± 0.034 | 0.919 ± 0.022 | 0.903 ± 0.042 | 0.790 ± 0.075 |
| **Entropy Sampling** | 0.927 ± 0.013 | 0.929 ± 0.014 | 0.928 ± 0.012 | 0.769 ± 0.103 |
| **Least Confidence** | 0.929 ± 0.012 | 0.931 ± 0.011 | 0.928 ± 0.013 | 0.804 ± 0.037 |
| **Mean STD** | 0.925 ± 0.015 | 0.930 ± 0.013 | 0.924 ± 0.016 | 0.802 ± 0.098 |
| **Margin Sampling** | 0.927 ± 0.006 | 0.929 ± 0.007 | 0.927 ± 0.005 | 0.787 ± 0.066 |
| **BatchBALD** | 0.933 ± 0.007 | 0.934 ± 0.009 | 0.933 ± 0.007 | 0.791 ± 0.071 |
| **Variational Ratios** | 0.927 ± 0.009 | 0.930 ± 0.010 | 0.927 ± 0.010 | 0.788 ± 0.076 |

Table 7: COVID Binary: Per-class metrics (mean ± std across seeds) when active learning algorithm terminated.

| **Acquisition Function** | **Precision C_0_** | **Precision C_1_** | **Sensitivity C_0_** | **Sensitivity C_1_** | **F1 C_0_** | **F1 C_1_** |
| --- | --- | --- | --- | --- | --- | --- |
| **Random** | 0.663 ± 0.152 | 0.961 ± 0.014 | 0.768 ± 0.091 | 0.925 ± 0.053 | 0.697 ± 0.086 | 0.942 ± 0.027 |
| **Entropy Sampling** | 0.753 ± 0.061 | 0.958 ± 0.019 | 0.738 ± 0.122 | 0.959 ± 0.018 | 0.738 ± 0.057 | 0.958 ± 0.007 |
| **Least Confidence** | 0.732 ± 0.065 | 0.964 ± 0.007 | 0.780 ± 0.043 | 0.952 ± 0.015 | 0.754 ± 0.039 | 0.958 ± 0.008 |
| **Mean STD** | 0.722 ± 0.085 | 0.964 ± 0.018 | 0.778 ± 0.117 | 0.947 ± 0.025 | 0.739 ± 0.052 | 0.955 ± 0.010 |
| **Margin Sampling** | 0.734 ± 0.041 | 0.961 ± 0.012 | 0.760 ± 0.078 | 0.954 ± 0.011 | 0.743 ± 0.030 | 0.957 ± 0.003 |
| **BatchBALD** | 0.763 ± 0.043 | 0.962 ± 0.013 | 0.763 ± 0.085 | 0.960 ± 0.013 | 0.759 ± 0.033 | 0.961 ± 0.004 |
| **Variational Ratios** | 0.735 ± 0.053 | 0.961 ± 0.014 | 0.762 ± 0.091 | 0.954 ± 0.017 | 0.743 ± 0.036 | 0.957 ± 0.006 |

Table 8: COVID Binary: Wilcoxon one-sided signed-rank p-values for each acquisition function vs Random.

| **Acquisition Function** | **Accuracy** | **F1 Macro** | **Precision Macro** | **Specificity Macro** | **AU-ROC** | **AU-PRC** |
| --- | --- | --- | --- | --- | --- | --- |
| **BatchBALD** | 0.021 | 0.037 | 0.255 | 0.074 | 0.086 | 0.250 |
| **Entropy Sampling** | 0.061 | 0.230 | 0.061 | 0.020 | 0.102 | 0.031 |
| **Least Confidence** | 0.014 | 0.109 | 0.003 | 0.040 | 0.001 | 0.109 |
| **Margin Sampling** | 0.005 | 0.207 | 0.009 | 0.102 | 0.258 | 0.091 |
| **Mean STD** | 0.144 | 0.119 | 0.004 | 0.455 | 0.181 | 0.152 |
| **Var Ratios** | 0.016 | 0.037 | 0.029 | 0.125 | 0.116 | 0.062 |

Table 9 COVID Multi: Macro metrics (mean ± std across seeds) when active learning algorithm terminated.

| **Acquisition Function** | **Accuracy** | **Macro F1** | **Macro Precision** | **Macro Sensitivity** | **Macro Specificity** | **AU-ROC** | **AU-PRC** |
| --- | --- | --- | --- | --- | --- | --- | --- |
| **Random** | 0.687 ± 0.028 | 0.677 ± 0.029 | 0.705 ± 0.039 | 0.676 ± 0.035 | 0.819 ± 0.015 | 0.849 ± 0.019 | 0.751 ± 0.034 |
| **Entropy Sampling** | 0.711 ± 0.021 | 0.710 ± 0.021 | 0.716 ± 0.035 | 0.719 ± 0.019 | 0.838 ± 0.009 | 0.860 ± 0.017 | 0.767 ± 0.025 |
| **Least Confidence** | 0.695 ± 0.015 | 0.692 ± 0.024 | 0.702 ± 0.023 | 0.691 ± 0.031 | 0.824 ± 0.008 | 0.847 ± 0.008 | 0.743 ± 0.025 |
| **Mean STD** | 0.705 ± 0.022 | 0.702 ± 0.026 | 0.707 ± 0.034 | 0.704 ± 0.024 | 0.831 ± 0.010 | 0.850 ± 0.026 | 0.752 ± 0.044 |
| **Margin Sampling** | 0.704 ± 0.013 | 0.699 ± 0.012 | 0.706 ± 0.031 | 0.702 ± 0.018 | 0.834 ± 0.003 | 0.855 ± 0.011 | 0.750 ± 0.026 |
| **BatchBALD** | 0.699 ± 0.027 | 0.696 ± 0.020 | 0.709 ± 0.029 | 0.696 ± 0.029 | 0.831 ± 0.015 | 0.853 ± 0.020 | 0.756 ± 0.031 |
| **Variational Ratios** | 0.693 ± 0.011 | 0.694 ± 0.010 | 0.702 ± 0.018 | 0.695 ± 0.020 | 0.826 ± 0.006 | 0.848 ± 0.015 | 0.748 ± 0.021 |

Table 10: COVID Multi: Weighted metrics (mean ± std across seeds) when active learning algorithm terminated.

| **Acquisition Function** | **Weighted F1** | **Weighted Precision** | **Weighted Sensitivity** | **Weighted Specificity** |
| --- | --- | --- | --- | --- |
| **Random** | 0.678 ± 0.027 | 0.694 ± 0.023 | 0.687 ± 0.028 | 0.769 ± 0.031 |
| **Entropy Sampling** | 0.708 ± 0.022 | 0.717 ± 0.022 | 0.711 ± 0.021 | 0.804 ± 0.012 |
| **Least Confidence** | 0.690 ± 0.015 | 0.693 ± 0.014 | 0.695 ± 0.015 | 0.778 ± 0.015 |
| **Mean STD** | 0.701 ± 0.021 | 0.703 ± 0.022 | 0.705 ± 0.022 | 0.789 ± 0.015 |
| **Margin Sampling** | 0.702 ± 0.011 | 0.708 ± 0.010 | 0.704 ± 0.013 | 0.798 ± 0.014 |
| **BatchBALD** | 0.697 ± 0.025 | 0.708 ± 0.027 | 0.699 ± 0.027 | 0.793 ± 0.024 |
| **Variational Ratios** | 0.691 ± 0.009 | 0.695 ± 0.011 | 0.693 ± 0.011 | 0.784 ± 0.014 |

Table 11: COVID Multi: Per-class metrics (mean ± std across seeds) when active learning algorithm terminated.

| **Acquisition Function** | **Precision C_0_** | **Precision C_1_** | **Precision C_2_** | **Sensitivity C_0_** | **Sensitivity C_1_** | **Sensitivity C_2_** | **F1 C_0_** | **F1 C_1_** | **F1 C_2_** |
| --- | --- | --- | --- | --- | --- | --- | --- | --- | --- |
| **Random** | 0.769 ± 0.118 | 0.619 ± 0.071 | 0.728 ± 0.053 | 0.711 ± 0.117 | 0.511 ± 0.118 | 0.808 ± 0.112 | 0.726 ± 0.068 | 0.548 ± 0.057 | 0.758 ± 0.037 |
| **Entropy Sampling** | 0.750 ± 0.095 | 0.622 ± 0.046 | 0.776 ± 0.033 | 0.789 ± 0.120 | 0.599 ± 0.107 | 0.769 ± 0.076 | 0.757 ± 0.040 | 0.603 ± 0.053 | 0.769 ± 0.030 |
| **Least Confidence** | 0.764 ± 0.058 | 0.608 ± 0.029 | 0.735 ± 0.025 | 0.737 ± 0.118 | 0.551 ± 0.073 | 0.786 ± 0.077 | 0.746 ± 0.077 | 0.575 ± 0.036 | 0.757 ± 0.029 |
| **Mean STD** | 0.747 ± 0.081 | 0.628 ± 0.036 | 0.745 ± 0.024 | 0.760 ± 0.056 | 0.551 ± 0.057 | 0.800 ± 0.056 | 0.750 ± 0.052 | 0.585 ± 0.030 | 0.770 ± 0.020 |
| **Margin Sampling** | 0.735 ± 0.102 | 0.616 ± 0.028 | 0.767 ± 0.029 | 0.740 ± 0.097 | 0.592 ± 0.059 | 0.775 ± 0.068 | 0.727 ± 0.029 | 0.601 ± 0.022 | 0.768 ± 0.022 |
| **BatchBALD** | 0.750 ± 0.071 | 0.609 ± 0.054 | 0.767 ± 0.050 | 0.723 ± 0.098 | 0.608 ± 0.100 | 0.758 ± 0.105 | 0.729 ± 0.041 | 0.601 ± 0.041 | 0.757 ± 0.046 |
| **Variational Ratios** | 0.759 ± 0.063 | 0.600 ± 0.032 | 0.746 ± 0.028 | 0.746 ± 0.087 | 0.574 ± 0.073 | 0.764 ± 0.055 | 0.747 ± 0.033 | 0.583 ± 0.026 | 0.753 ± 0.018 |

Table 12: COVID Multi: Wilcoxon one-sided signed-rank p-values for each acquisition function vs Random.

| **Acquisition Function** | **Accuracy** | **F1 Macro** | **Precision Macro** | **Specificity Macro** | **AU-ROC** | **AU-PRC** |
| --- | --- | --- | --- | --- | --- | --- |
| **BatchBALD** | 0.116 | 0.372 | 0.102 | 0.146 | 0.510 | 0.144 |
| **Entropy Sampling** | 0.094 | 0.256 | 0.194 | 0.020 | 0.568 | 0.500 |
| **Least Confidence** | 0.093 | 0.035 | 0.209 | 0.248 | 0.254 | 0.340 |
| **Margin Sampling** | 0.216 | 0.131 | 0.850 | 0.029 | 0.795 | 0.422 |
| **Mean STD** | 0.076 | 0.475 | 0.475 | 0.248 | 0.606 | 0.445 |
| **Var Ratios** | 0.455 | 0.777 | 0.435 | 0.590 | 0.936 | 0.746 |

Figure 1: Binary classification results across various macro metrics as a function of the number of imaging data samples acquired: Accuracy (a), AU-ROC (b), AU-PRC (c), Sensitivity (d), F1 (e), Specificity (f), Precision (g), NLL (h). The results are reported as [25th, 50th, 75th] percentiles. The baseline is denoted as a dotted line in red.

Figure 2: Multi-class classification results across various macro metrics as a function of the number of imaging data samples acquired: Accuracy (a), AU-ROC (b), AU-PRC (c), Sensitivity (d), F1 (e), Specificity (f), Precision (g), NLL (h). The results are reported as [25th, 50th, 75th] percentiles. The baseline is denoted as a dotted line in red.

Figure 3: Binary classification error comparison between Least Confidence and Random Sampling using only 9.24% of the imaging data samples across various metrics: Misclassification Rate (a), AU-ROC (b), AU-PRC (c), Sensitivity (d), F1 (e), Specificity (f), Precision (g), NLL (h).

Figure 4: Multi-class classification error comparison between Mean STD and Random Sampling using only 21.87% of the imaging data samples across various metrics: Misclassification Rate (a), AU-ROC (b), AU-PRC (c), Sensitivity (d), F1 (e), Specificity (f), Precision (g), NLL (h).

Figure 5: Mean and standard deviation of class distribution (in %) for binary (a) and multi-class (b) classification across various acquisition functions.

Figure 6: Mean and standard deviation of training and acquisition batch time for binary (a) and multi-class (b) classification across various acquisition functions.
